# Time course efficiency of MICE and HIIE on inhibitory control and HRV in adolescents with obesity and different cardiorespiratory fitness

**DOI:** 10.3389/fpsyg.2023.1242190

**Published:** 2023-08-17

**Authors:** Zhan-Tao Feng, Zhi-Xiong Mao, Feng-Bo Liu, Xiao-Wei Ou

**Affiliations:** ^1^School of Psychology, Beijing Sport University, Beijing, China; ^2^College of Sports and Health, Shandong Sport University, Rizhao, China; ^3^School of Physical Education, Zhengzhou University of Light Industry, Zhengzhou, China

**Keywords:** moderate-intensity continuous exercise, high-intensity interval exercise, inhibitory control, HRV, cardiorespiratory fitness, obese adolescents

## Abstract

**Background:**

Adolescent obesity is associated with impaired inhibitory control. Acute exercise can improve executive function. However, due to the influence of exercise intensity, cognitive test timing, and cardiorespiratory fitness (CF) level, the most effective exercise program remains controversial.

**Methods:**

The current study investigated the time-course effects of moderate-intensity continuous exercise (MICE) and high-intensity interval exercise (HIIE) on inhibitory control (Stroop) and task-related heart rate variability (HRV) in adolescents with different CF. A mixed experimental design of 2 CF levels (high CF, HCF; low CF, LCF) × 3 exercise methods (MICE, HIIE, CON) × 3 test timing (pre, post-0, post-20) was adopted. Heart rate variability (HRV) and Stroop task tests were conducted before exercise (pre), immediately after exercise (post-0), and 20 min after exercise (post-20).

**Results:**

Individuals with HCF exhibited a positive decrease in Stroop response time immediately and 20 min after MICE and HIIE, compared to pretest response times (RT). Conversely, individuals with LCF showed a slight increase in Stroop task (RT) only immediately after HIIE. All individuals had a slight increase in ACC after MICE and HIIE compared to before exercise. In addition, compared with the control group, the time-domain index (the square root of the mean squared differences of successive NN intervals, RMSSD) of HRV was significantly decreased, the frequency-domain index (the absolute power of the Low-Frequency band/the absolute power of the High-Frequency band ratio, LF/HF) was significantly increased after MICE and HIIE, and the effect of HIIE on RMSSD and LF/HF was significantly greater than that of MICE.

**Conclusion:**

The current study found that the acute effects of MICE and HIIE on inhibitory control in obese adolescents were influenced by the interaction of cognitive test timing and cardiorespiratory fitness. Individuals with high cardiorespiratory fitness performed better on the Stroop task than individuals with low cardiorespiratory fitness. The inhibitory control of HIIE in high-cardiorespiratory obese adolescents produced positive effects similar to those in MICE but more lasting, suggesting that HIIE is more beneficial for high-cardiorespiratory obese adolescents. MICE promoted inhibitory control in obese adolescents with low cardiorespiratory fitness, but HIIE impaired inhibitory control in obese adolescents with low cardiorespiratory fitness immediately after exercise, suggesting that low cardiopulmonary fitness obese adolescents may be suitable for MICE rather than HIIE exercise intervention. The shift from balanced HRV to sympathetic dominance after acute exercise reflects increased arousal levels and may be one of the underlying mechanisms by which acute exercise brings benefits to executive function.

## 1. Introduction

In recent years, the obesity epidemic among adolescents has been on the rise globally. China is no exception, becoming the country with the largest number of adolescents with obesity in the world (Zhang and Ma, 2017). Adolescent obesity not only affects normal growth and development (Wu et al., 2017) but is also associated with an increased risk of impaired cognitive function (Hsu et al., 2015; Sweat et al., 2017), especially executive function (Tee et al., 2018; Yang et al., 2018). This may be related to the significantly lower cerebral blood flow velocity and cerebral blood flow in obese adolescents than in normal-weight adolescents (Doroszewska et al., 2000). In addition, physiological changes such as inflammation and reduced insulin and dopamine levels caused by obesity are also major potential mechanisms for obesity-induced cognitive decline (Cui and Li, 2016). The period of childhood and adolescence is a sensitive period for significant changes in brain morphology and function, as well as a golden period for cognitive function development (Spear, 2013). Environmental factors are particularly sensitive to the effects of the brain and behavior during this period (Marco et al., 2011). Inhibition control is a determinant of academic success (Diamond, 2013). Therefore, it is of great significance to find a way to enhance and improve inhibitory control in adolescents with obesity.

Inhibitory control, as the main component of executive function, refers to the ability of individuals to control their attention, behavior, thoughts, and/or emotions to overcome internal dominant responses and external temptations, thus avoiding inappropriate behaviors (Diamond, 2013). According to Diamond’s definition, inhibition control can be divided into interference control (inhibition of interference from irrelevant competing stimuli) and response inhibition (inhibition of behavioral responses that do not meet current needs) (Diamond, 2013).

Physical exercise, as a healthy and non-invasive intervention, may provide an important target to decrease the obesity of adolescents and restore and improve the poor cognitive function observed in adolescents with obesity (Xie et al., 2017). The improvement of cognitive function by acute exercise has been supported by cross-sectional studies (e.g., Hillman et al., 2006), longitudinal studies (e.g., Sofi et al., 2011), and meta-analysis (e.g., Moreau and Chou, 2019). However, the most effective exercise program remains controversial due to the influence of differences in exercise intensity, cognitive testing time points, and individual differences in cardiorespiratory fitness (CF) (Pontifex et al., 2019).

According to the inverted U hypothesis (Yerkes and Dodson, 1908), most of the previous studies on improving cognition with acute exercise focused on moderate-intensity continuous exercise (MICE), especially in tasks requiring improved inhibitory control (O’Leary et al., 2011). However, compared with MICE, high-intensity interval exercise (HIIE) induces physiological adaptation (Benda et al., 2015; Gayda et al., 2016), cognitive improvement (Biddle and Batterham, 2015; Hötting et al., 2016; Hillman and Biggan, 2017), and maybe a more promising exercise program. Tsukamoto et al. (2016), used a 4 min high-intensity cycling and 3 min HIIE regimen in young adults. They showed that both MICE and HIIE improved Stroop task performance compared to baseline, but that the improvement in inhibition control with HIIE lasted longer than with MICE. Similarly, Kao et al. (2017) compared the effects of 9 min of HIIE and 20 min of MICE on inhibitory control (Flanker) in young people, and Lambrick et al. (2016) compared the effects of 15 min of MICE and HIIE on children’s inhibitory control (Stroop). Based on the characteristics of paroxysmal, high-intensity, and intermittent exercise of adolescents (Bailey et al., 1995), short-term high-intensity exercise is more natural, more attractive, and easier to continue than traditional moderate-intensity exercise (Buchan et al., 2013). Additionally, based on the positive effects of HIIE on cognitive function and mental health of healthy-weight adolescents, HIIE may also be effective in improving cognitive function in adolescents with obesity (Deng and Cao, 2019).

The influence of exercise intensity may depend on the individual’s tolerance to exercise stimuli. Individuals with higher cardiorespiratory fitness may be more accustomed to higher workloads (Hüttermann and Memmert, 2014), and higher levels of fitness have been associated with enhancements in brain structure, brain functioning, and cognitive performance (McAuley et al., 2004; Voss et al., 2011). Therefore, it is important to consider individual differences in CF when studying the cognitive effects of different levels of physical exercise. A meta-analysis by Chang et al. (2012) showed that, compared with individuals with low or moderate CF, only individuals with high CF obtained the greatest acute exercise-cognitive benefits in both the immediate and delayed stages after exercise. Hogan et al. (2015) explored the effects of 20 min of MICE on adolescent inhibitory control (Flanker) and Chu et al. (2015) explored the effects of 20 min of MICE on inhibitory control in older adults (Stroop). Individuals with high CF showed greater improvement in inhibitory control after exercise than individuals with low CF. In contrast, several studies assessing working memory (Li et al., 2019) and inhibitory control (Cui et al., 2020) after acute exercise in young adults with high and low cardiorespiratory fitness have only found that low-health individuals benefit from acute exercise. Due to differences in the existing literature on factors such as individual age and cognitive task tests, the role of cardiorespiratory fitness in MICE and HIIE improved inhibitory control is unclear.

The underlying hypothesis for the effects of acute exercise on cognition is the physiological changes produced by exercise (Ferris et al., 2007). Additionally, recovery from exercise-induced physiological changes occurs gradually (Audiffren and Andre, 2015); therefore, temporal differences in the effects of acute exercise on cognitive function may occur. Mekari et al. (2015) found that after high-intensity exercise, Stroop performance decreased during and immediately after exercise, while Stroop performance improved for all participants 15 min after low-, moderate-, and high-intensity exercise. Lambrick et al. (2016) compared the effects of 15 min of MICE and HIIE on inhibitory control (Stroop) in children and found that both MICE and HIIE improved inhibitory control immediately after exercise, but only HIIE demonstrated improvement 30 min after exercise. Cooper et al. (2018) studied the effects of 60 min of basketball-based HIIE on inhibitory control (Stroop) and working memory (Sternberg) in adolescents and found that HIIE improved inhibitory control and working memory performance, both immediately and 45 min after exercise. These studies suggest that the potential cognitive benefits of acute exercise are influenced by the interaction between exercise intensity and cognitive testing time points. A meta-analysis by Chang et al. (2012) showed enhancement in cognitive tasks performed immediately after exercise (low or moderate intensity); however, after a 1 min delay, very low-intensity exercise no longer had a positive effect, while more intense exercise (high-intensity) continued to increase cognitive performance.

Exercise is a stressor that promotes arousal by increasing the expression of catecholamines (Tsukamoto et al., 2016), thereby improving cognitive performance (Anish, 2005). Heart rate variability (HRV) is an indicator of autonomic nervous system function, and to some extent, measures the level of arousal of individuals (Weissman et al., 2018). Generally speaking, HRV decreases with the increase in arousal (Zhong et al., 2005). In terms of the HRV frequency domain, the absolute power of the Low-Frequency band (LF) is mainly an indicator of sympathetic influence (accompanied by a parasympathetic component). While the absolute power of the High-Frequency band (HF) is considered to be a marker of parasympathetic influence (Shaffer et al., 2014; Michael et al., 2017). The LF/HF ratio reflects the relative activity of sympathetic and parasympathetic nerves, with a low ratio reflecting greater parasympathetic nerve activity (Shaffer et al., 2014). In terms of the time domain of HRV, the square root of the mean squared differences of successive NN intervals (RMSSD) is the main index reflecting vagal changes in HRV estimation (Shaffer et al., 2014).

Physical exercise primarily activates the left dorsolateral prefrontal cortex (l-DLPFC) to enhance executive function, which is the location of the brain involved in inhibitory control (Byun et al., 2014; Kujach et al., 2018). Based on the neurovisceral integration hypothesis (Thayer et al., 2009), changes in HRV are associated with executive function and can serve as an indicator of the activation of prefrontal neural structures (Dum et al., 2016). Ahern et al. (2001) have found that inactivation of the prefrontal cortex is associated with decreased HRV. Elevated HRV caused by parasympathetic inhibition (Mathewson et al., 2010) or sympathetic excitation (Murray and Russoniello, 2012) after acute exercise is associated with better performance of the executive function. Therefore, the current study evaluated changes in HRV at both immediate and delayed stages after different intensity exercises to provide new insights into the time course efficiency of exercise intensity on task-related HRV and inhibition control.

This study aimed to explore the time-course effects of traditional MICE and emerging HIIE on inhibitory control and HRV in adolescents with obesity and different levels of CF. Based on previous studies (Chang et al., 2012), we hypothesized that both MICE and HIIE would improve inhibitory control in adolescents with obesity immediately after exercise, and HIIE would produce better results than MICE in the delayed post-exercise phase, compared with the non-exercising control group (Tsukamoto et al., 2016). Furthermore, we hypothesized that the influence of MICE and HIIE on inhibitory control would be moderated by the level of CF (Brisswalter et al., 2002). Additionally, regarding the measurement of HRV, the time-domain index RMSSD would decrease, while the frequency-domain index LF/HF ratio would increase after exercise.

## 2. Materials and methods

### 2.1. Subjects

According to the Overweight and Obesity BMI Screening Criteria for Chinese Students (Ji, 2004), a total of 120 adolescents with obesity (BMI ≥ 25.7 kg/m^2^), aged 12–14 years old, were randomly selected using a body composition measuring instrument (GAIA KIKO, Korea). Of these, 45 people in the high CF group (High CF, HCF) and 45 people in the low CF group (Low CF, LCF) were randomly divided into three groups according to CF level, 30 people in the MICE group (15 people in HCF, 15 people in LCF people), 30 people in the HIIE group (HCF 15 people, LCF 15 people), and 30 people in the inactive control group (CON; HCF 15 people, LCF 15 people). All subjects met the following inclusion criteria: (1) normal or corrected-to-normal vision; (2) no color blindness; (3) no history of neurological disorders or cardiovascular disease; (4) right-handed (*Edinburgh Handedness Questionnaire*); and (5) a passing score on the *Physical Activity Readiness Questionnaire* (PAR-Q) to assesses the ability to complete CF tests. Written informed consent was obtained from the subjects and their legal guardians, and the research protocol was approved by the Ethics Committee of Beijing Sport University.

### 2.2. Experimental design

A 2 CF levels (HCF, LCF) × 3 exercise methods (MICE, HIIE, CON) × 3 test timing (before exercise = pre, immediately after exercise = post-0, and 20 min after exercise = post-20) mixed experimental design was used. CF level and exercise method were the between-group variables, and the test timing was the within-group variable. Reaction time (RT) and accuracy rate (ACC) of the Stroop task, and the time-domain (RMSSD) and frequency-domain (LF/HF) indices of HRV were the dependent variables.

### 2.3. Testing and tools

#### 2.3.1. CF test

Based on the safety of the test process and considering the abilities of adolescents with obesity, the submaximal intensity YMCA cycle ergometer (Monark 839E, Sweden) test program was selected to complete the CF evaluation [VO_2max_ (mL/kg/min); Beekley et al., 2004]. CF was rated as very poor, poor, fair, average, good, very good, or excellent. In this study, subjects with good, very good, or excellent CF were included in the HCF group, and subjects with very poor, poor, or fair CF were included in the LCF group (Oberste et al., 2019).

#### 2.3.2. Inhibition control test (Stroop)

All Stroop task evaluations were performed in the laboratory using E-Prime 3.0. First, a white fixation point (+symbol) was displayed in the center of a black computer screen for 500 milliseconds. Then 1,500 milliseconds of color word stimulus was presented randomly after a 300 or 500 millisecond fixation point interval. Color word stimulus consisted of different color words (“red, “yellow,” “green,” and “blue”) presented on a black screen, and was divided into two conditions: congruent and incongruent. The congruent condition occurred when the word matched the font color; for example, when the word “red” was written in red. The incongruent condition occurred when the word did not match the font color; for example, the word “red” was written in green. The number of congruent trials and incongruent trials was 1:1. Subjects were required to judge the color words and respond quickly and accurately to the keys (red “D,” yellow “F,” green “J,” blue “K”). After the subject pressed the key, the trial ended and the next trial began.

The whole Stroop task includes two parts: practice and formal experiment. The practice consisted of 24 trials, while the formal experiment consisted of 96 trials. Practice experiments provided correct and incorrect feedback information, while the formal experiment trials provided no feedback information. The formal experiment was divided into two blocks, with each block containing 48 trials. There was a 30 s interval between the two blocks.

#### 2.3.3. HRV test

An HRV measuring instrument (mega, eMotion HRV 3D, Finland) and HRV-Scanner software were used for HR recording and HRV analysis. The eMotion HRV 3D testing instrument automatically measures the activity of the autonomic nervous system, reflecting the balance of the sympathetic and parasympathetic branches. Several parameters related to heart rate variability were obtained, including time domain indicators (RMSSD) and frequency domain indicators (LF, HF, LF/HF).

### 2.4. Exercise intervention methods

Studies have found that, in the exercise-cognitive task continuation paradigm, compared with running, cycling has a more significant effect on improving cognitive performance (Lambourne and Tomporowski, 2010). Therefore, both MICE and HIIE in the current study adopted the Bicycle Ergometer Custo Med GmbH ec3000 (United Kingdom). During the exercise, according to the exercise intensity classification standard of Norton et al. (2010), heart rate reserve (HRR) was used to determine exercise intensity, HRR = [(HRmax − HRrest) × %target intensity] + HRrest, low intensity (20–40% HRR), medium intensity (41–60%HRR), high intensity (61–85%HRR).

**Figure 1:**
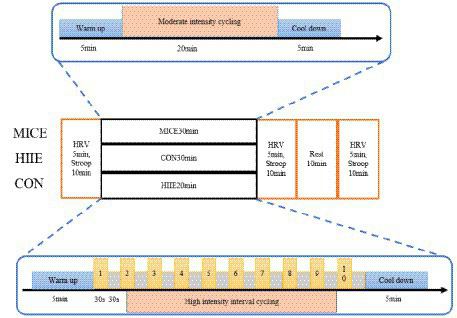
Flow chart of the testing protocol.

According to the principle of matching energy consumption (kcal) between MICE and HIIE, the exercise program (Figure 1) was conducted as follows:

*1. MICE group: 5 min of low-intensity warm-up, 20 min of moderate-intensity continuous cycling, and 5 min of relaxation, totaling 30 min.*

*2. HIIE group: 5 min of low-intensity warm-up, 10 rounds of 30 s: 30 s high-intensity exercise intermittent cycling, 5 min of low-intensity relaxation, totaling 20 min.*

*3. CON group. watch a 30 min neutral video.*

Participants wore a Polar H10 heart rate strap (Polar, Kemple, Finland) before each workout and until the end of the workout. Rating of perceived exercise (RPE) was assessed every 5 min throughout the HIIE and MICE exercises.

### 2.5. Experimental procedure

Participants first filled out the informed consent forms and demographic information was collected, followed by CF test and Stroop task practice. To eliminate the effect of exercise during the CF test, MICE, HIIE, and CON exercises were performed in the three groups 1 week later, and HRV and Stroop’s tasks were evaluated before, immediately after, and 20 min after exercise.

### 2.6. Statistical analyses

Collected behavioral data was imported into Excel for preprocessing. First, subjects whose correct rate was less than 80% and whose response time was less than 200 milliseconds in the Stroop task were eliminated. Finally, response time and accuracy data beyond ±3 SD were eliminated.

The preprocessed data were analyzed in SPSS 26.0, and the normal distribution of the data was verified by the Shapiro–Wilk normality test. The HR before and after exercise was subjected to a two-factor repeated measure analysis of variance (ANOVA) of the 3 exercise groups (MICE, HIIE, CON) × 3 test timings (pre, post-0, post-20). The response time and accuracy on the Stroop task, as well as the RMSSD and LF/HF of HRV, were, respectively, evaluated for the 2 CF levels (HCF, LCF) × 3 exercise groups (MICE, HIIE, CON) × 3 test timing (pre, post-0, post-20) for three-factor repeated measures ANOVA. Spherical data were analyzed using the Mauchly test, and non-spherical data were analyzed with the Greenhouse–Geisser correction. If ANOVA showed that the second or third-order interaction was significant, a simple-effects analysis was performed, with the significance level set at *p* < 0.05.

## 3. Results

### 3.1. Demographic variables and exercise parameters

#### 3.1.1. Demographic variables

An independent sample *t*-test was used for demographic variables and showed that there were significant differences between each group in CF (VO_2max_; *p*s < 0.05); however, there were no significant differences in age, height, weight, or BMI (*p*s > 0.05; see Table 1).

**Table 1:** Descriptive statistics of demographic variables and exercise parameters.

Index	MICE		HIIE		CON	
	L-fitness	H-fitness	L-fitness	H-fitness	L-fitness	H-fitness
Age (years)	13.07 ± 0.59	13.13 ± 0.64	13.07 ± 0.59	13.40 ± 0.63	13.17 ± 0.49	13.20 ± 0.48
Weight (kg)	76.84 ± 9.27	74.19 ± 7.88	76.65 ± 8.18	74.51 ± 10.32	77.32 ± 10.26	74.71 ± 9.11
Height (m)	1.66 ± 0.08	1.64 ± 0.05	1.63 ± 0.08	1.66 ± 0.07	1.66 ± 0.08	1.66 ± 0.07
BMI	28.49 ± 2.78	27.69 ± 2.51	28.76 ± 2.99	28.00 ± 2.21	28.58 ± 2.71	28.38 ± 2.66
VO_2max_ (ml/kg/min)	37.72 ± 4.96	50.80 ± 4.78	37.03 ± 4.96	49.537 ± 3.50	36.93 ± 4.28	49.60 ± 4.19
HRmax (bpm)	162.47 ± 5.78	161.07 ± 5.36	178.80 ± 6.20	181.00 ± 6.93	86.75 ± 2.84	81.37 ± 5.67
HRav (bpm)	147.27 ± 4.20	148.20 ± 3.39	158.93 ± 4.51	159.53 ± 4.66	79.54 ± 3.24	76.58 ± 3.38
RPEav	12.64 ± 0.48	12.36 ± 0.52	14.86 ± 0.44	14.21 ± 0.48	9.64 ± 0.67	9.61 ± 0.61

#### 3.1.2. Exercise parameters

**Figure 2:**
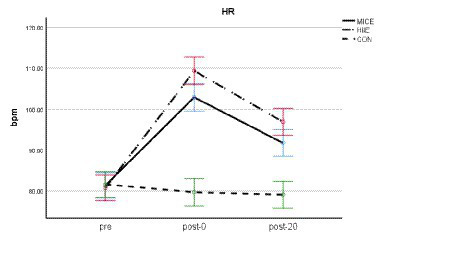
HR analyses of three groups of subjects before and after exercise.

Table 1 shows the maximum heart rate (HRmax), average heart rate (HRav), and average RPE (RPEav) data of the MICE and HIIE groups. All subjects met the requirements of exercise intensity. A two-factor repeated measures ANOVA of HR before and after exercise showed that the main effect of test timing was significant [*F* (2, 82) = 256.725, *p* < 0.001, *η*_p_^2^ = 0.857], the main effect of exercise methods was significant [*F* (2, 87) = 37.022, *p* < 0.001, *η*_p_^2^ = 0.460], and the interaction between test timing and exercise methods was also significant [*F* (4, 174) = 25.101, *p* < 0.001, *η*_p_^2^ = 0.366]. Further simple effect analysis showed that there were significant differences in HR between pre, post-0, and post-20 in MICE and HIIE (*p*s < 0.001), post-0 > post-20 > pre. There were no significant differences in HR between pre, post-0, and post-20 in CON (*p*s > 0.05; see Figure 2).

### 3.2. Stroop data

The RT and ACC analyses of the Stroop task found that the RT of the congruent condition in each test was significantly lower than that of the incongruent condition (*p*s < 0.001), and the ACC of the congruent condition was significantly higher than that of the incongruent condition (*p* < 0.05), indicating a significant Stroop effect. In addition, the RT of participants with HCF in the three groups was significantly lower than that of participants with LCF (*p*s < 0.01); however, there was no significant difference observed in ACC between the two groups (*p*s > 0.05; see Table 2).

**Table 2:** Descriptive statistics of Stroop task RT and ACC (M ± SD).

Condition	Method	CF	pre-RT (ms)	post-0RT (ms)	post-20RT (ms)	pre-ACC (%)	post-0ACC (%)	post-20ACC (%)
Congruent	MICE	LCF	630.22 ± 58.60	590.07 ± 50.14	607.08 ± 44.38	93.06 ± 3.46	94.81 ± 2.99	94.72 ± 3.58
		HCF	594.30 ± 73.14	560.35 ± 70.43	552.42 ± 73.12	94.07 ± 4.71	94.99 ± 3.94	95.63 ± 3.22
	HIIE	LCF	621.96 ± 71.01	640.60 ± 70.08	619.57 ± 68.82	93.67 ± 3.63	95.44 ± 3.46	94.81 ± 3.46
		HCF	590.70 ± 74.65	549.29 ± 65.83	551.55 ± 64.10	94.38 ± 3.32	96.11 ± 3.02	96.33 ± 2.82
	CON	LCF	625.6 ± 80.37	620.60 ± 75.93	623.07 ± 68.66	94.78 ± 3.93	94.81 ± 3.12	95.28 ± 3.27
		HCF	584.53 ± 77.68	579.00 ± 71.28	580.13 ± 77.27	94.29 ± 4.01	95.76 ± 3.18	94.30 ± 4.32
Incongruent	MICE	LCF	709.91 ± 63.07	653.43 ± 64.89	663.58 ± 50.36	91.26 ± 3.75	93.05 ± 3.45	93.05 ± 4.01
		HCF	653.01 ± 78.07	600.45 ± 78.94	599.08 ± 77.46	91.11 ± 4.13	92.04 ± 3.69	93.92 ± 3.19
	HIIE	LCF	705.58 ± 78.47	724.53 ± 68.64	676.82 ± 61.40	90.56 ± 3.74	91.28 ± 3.79	92.69 ± 3.74
		HCF	648.89 ± 81.73	603.41 ± 75.09	612.49 ± 75.81	92.13 ± 2.76	92.31 ± 3.52	93.50 ± 3.15
	CON	LCF	710.73 ± 80.91	707.93 ± 75.03	703.13 ± 82.92	91.38 ± 3.61	92.03 ± 3.77	92.39 ± 4.07
		HCF	641.87 ± 73.99	635.67 ± 77.44	638.27 ± 75.92	91.65 ± 3.89	92.44 ± 3.68	92.68 ± 3.73

#### 3.2.1. RT of congruent Stroop condition

**Figure 3:**
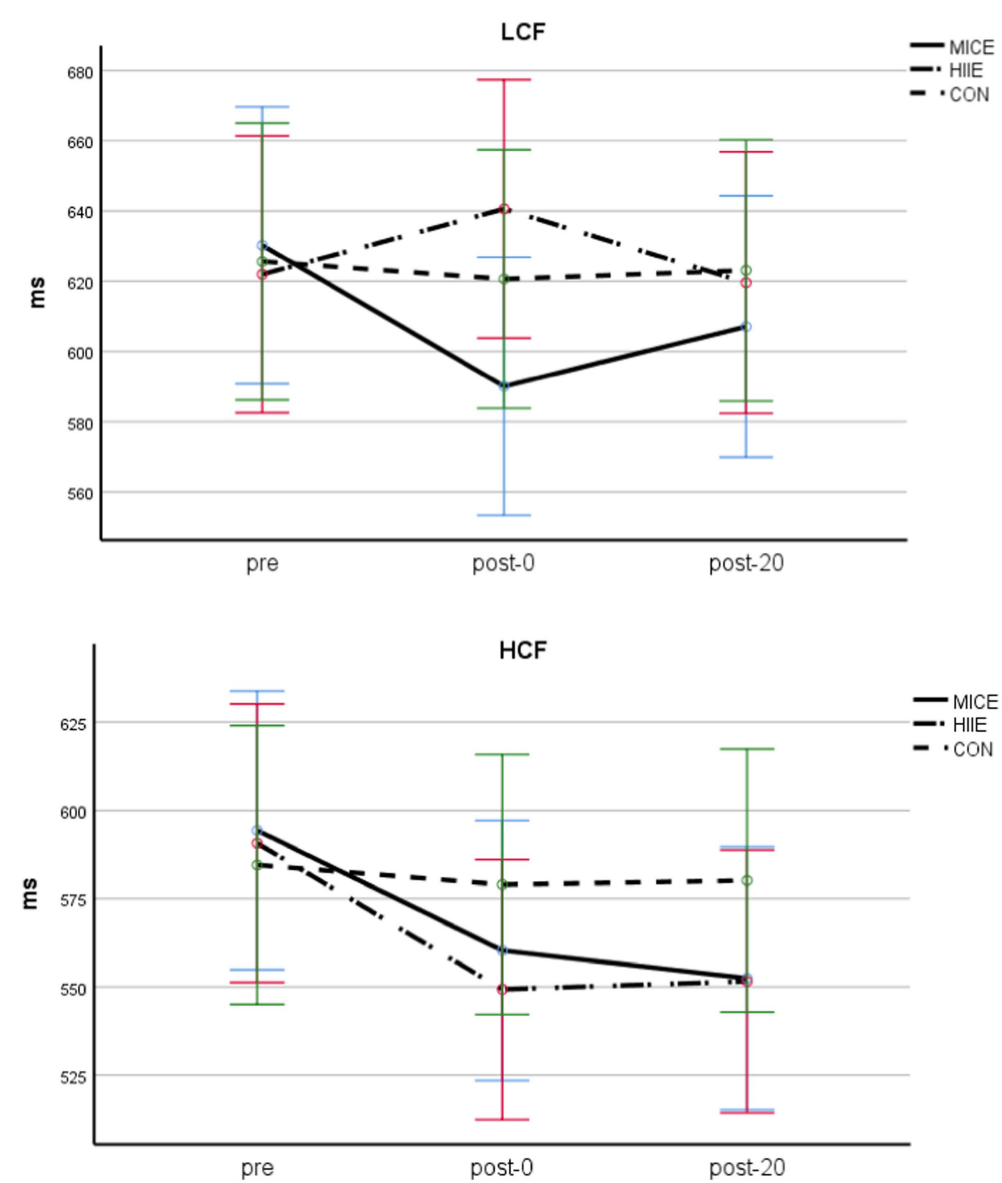
Congruent condition RT in adolescents with obesity and different CF.

A three-factor repeated measures ANOVA for congruent condition RT was performed (see Table 2 and Figure 3). The main effects of test timing [*F* (2, 84) = 11.911, *p* < 0.001, *η*_p_^2^ = 0.124] and CF level [*F* (1, 84) = 10.888, *p* < 0.01, *η*_p_^2^ = 0.115] were significant. Additionally, the interaction between test timing and exercise method was significant [*F* (4, 84) = 3.229, *p* < 0.05, *η*_p_^2^ = 0.071], the interaction between test timing and CF level was marginally significant (*p* = 0.050), and the interaction between test timing, exercise methods, and CF level was significant [*F* (1, 84) = 3.328, *p* < 0.05, *η*_p_^2^ = 0.072]. Further simple effect analyses showed that, for the LCF group, there were significant differences between pre and post-0 of MICE (*p* < 0.01), but post-20 exhibited no significant difference from pre and post-0 (*p*s > 0.05).

There was no significant difference between pre, post-0, and post-20 of HIIE (*p*s > 0.05); however, it can be seen from the mean that post-0 is higher than pre and post-20. For the HCF group, both MICE and HIIE showed significant differences between post-0, post-20, and pre (*p*s < 0.05), but there was no significant difference between post-0 and post-20 (*p*s > 0.05). In the CON group, there was no significant difference between the two groups in pre, post-0 and post-20(*p*s > 0.05). Finally, other main and interaction effects were not significant (*p*s > 0.05).

#### 3.2.2. RT of incongruent Stroop condition

**Figure 4:**
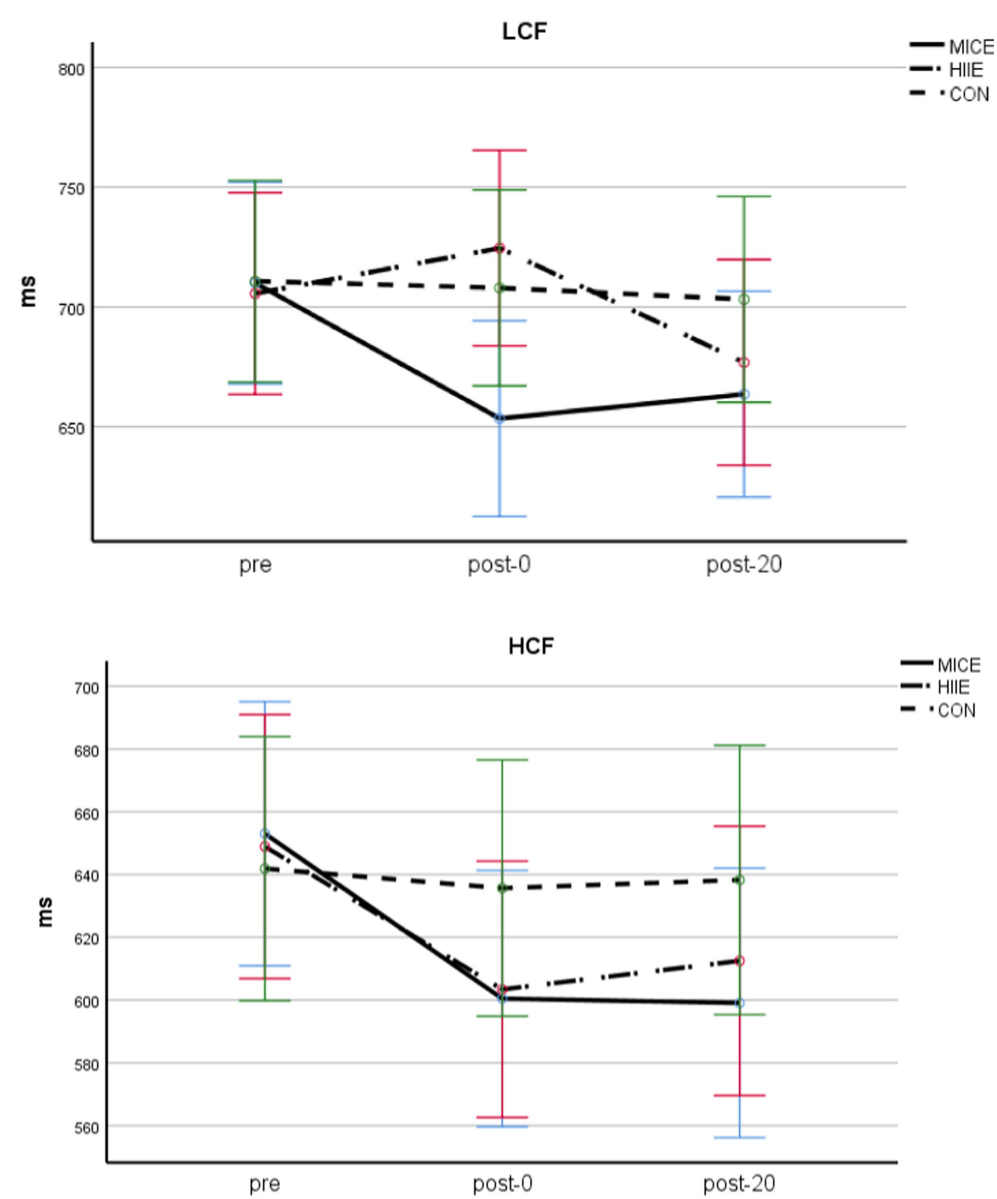
Incongruent condition RT in adolescents with obesity and different CF.

A three-factor repeated measures ANOVA for incongruent condition RT (see Table 2 and Figure 4) showed that the main effect of test timing [*F* (2, 84) = 17.825, *p* < 0.001, *η*_p_^2^ = 0.175] and CF level [*F* (1, 84) = 18.406, *p* < 0.001, *η*_p_^2^ = 0.180] was significant, the interaction of test timing and exercise methods was significant [*F* (4, 84) = 5.519, *p* < 0.001, *η*_p_^2^ = 0.116], and the interaction between test timing, exercise methods, and CF level was also significant [*F* (4, 84) = 2.716, *p* < 0.05, *η*_p_^2^ = 0.061]. Further simple effect analyses showed that, for LCF, there were significant differences between pre and post-0, as well as pre and post-20 of MICE (*p*s < 0.001), but no significant difference between post-0 and post-20 (*p* > 0.05); additionally, there was no significant difference between pre and post-0 of HIIE (*p* > 0.05), but post-20 was significantly different from both pre (*p* < 0.05) and post-0 (*p* < 0.005).

For HCF, both MICE and HIIE exercise methods exhibited significant differences between pre and post-0, as well as pre and post-20 (*p*s < 0.01); however, there was no significant difference between the post-0 and post-20 (*p*s > 0.05). In the CON group, there was no significant difference between the two groups in the pre, post-0, and post-20 comparisons (*p*s > 0.05). Other main and interactions effects were not significant (*p*s > 0.05).

#### 3.2.3. Stroop ACC

A three-factor repeated measures ANOVA was performed for ACC under both the congruent and incongruent conditions. For congruent condition ACC, only the main effect of test timing was significant [*F* (2, 84) = 5.648, *p* < 0.005, *η*_p_^2^ = 0.063]. Further *post hoc* comparisons found that there were significant differences between pre and post-0 (*p* < 0.01), as well as pre and post-20 after exercise (*p* < 0.05), but there was no significant difference between post-0 and post-20 after exercise (*p* > 0.05). For the incongruent condition ACC, only the main effect of test timing was significant [*F* (2, 84) = 6.814, *p* < 0.001, *η*_p_^2^ = 0.075]. Further *post hoc* comparison found no significant difference between pre and post-0 (*p* > 0.05), a significant difference between pre and post-20 (*p* < 0.001), and a marginally significant difference between post-0 and post-20 (*p* = 0.055). Other main and interaction effects of ACC were not significant (*p*s > 0.05).

### 3.3. HRV data

Descriptive statistics of HRV time-domain indicators (RMSSD) and frequency-domain indicators (LF/HF) of adolescents with obesity and different CF are shown in Table 3.

**Table 3:** Descriptive statistics of RMSSD and LF/HF.

Index	MICE		HIIE		CON	
	L-fitness	H-fitness	L-fitness	H-fitness	L-fitness	H-fitness
pre-RMSSD (ms)	44.05 ± 22.91	39.81 ± 23.99	42.20 ± 25.36	40.91 ± 20.05	32.08 ± 17.20	33.81 ± 17.76
post-0RMSSD (ms)	12.15 ± 9.05	14.18 ± 10.53	8.83 ± 5.07	8.35 ± 5.81	36.98 ± 14.50	36.07 ± 12.73
post-20RMSSD (ms)	30.36 ± 21.59	26.09 ± 20.05	17.45 ± 6.06	16.74 ± 8.11	39.77 ± 14.05	40.16 ± 15.29
pre-LF (n.u.)	63.34 ± 12.52	62.35 ± 11.95	57.89 ± 17.62	58.98 ± 15.00	60.72 ± 19.28	64.21 ± 7.57
post-0LF (n.u.)	76.54 ± 13.61	76.91 ± 10.64	78.09 ± 11.43	82.13 ± 10.34	66.36 ± 13.31	68.78 ± 13.97
post-20LF (n.u.)	56.36 ± 22.12	67.29 ± 21.33	67.35 ± 15.31	77.03 ± 10.63	60.79 ± 15.11	63.23 ± 10.32
pre-HF (n.u.)	36.66 ± 12.52	36.78 ± 11.12	37.25 ± 16.77	36.05 ± 12.84	32.45 ± 11.46	31.18 ± 10.30
post-0HF (n.u.)	22.21 ± 9.74	23.10 ± 10.64	19.24 ± 7.17	17.87 ± 10.33	42.05 ± 16.85	41.57 ± 16.69
post-20HF (n.u.)	25.72 ± 13.09	29.12 ± 11.58	27.91 ± 10.28	22.97 ± 10.63	38.75 ± 16.07	26.49 ± 10.30
pre-LF/HF	2.01 ± 0.94	1.91 ± 0.99	1.55 ± 0.72	1.94 ± 0.91	1.95 ± 0.47	2.52 ± 0.93
post-0LF/HF	4.19 ± 2.04	4.60 ± 2.03	4.55 ± 1.62	6.25 ± 2.52	1.65 ± 0.39	2.29 ± 1.22
post-20LF/HF	2.55 ± 1.27	3.35 ± 1.93	2.74 ± 1.10	4.25 ± 1.30	1.36 ± 0.30	1.98 ± 0.98

#### 3.3.1. HRV time domain index

**Figure 5:**
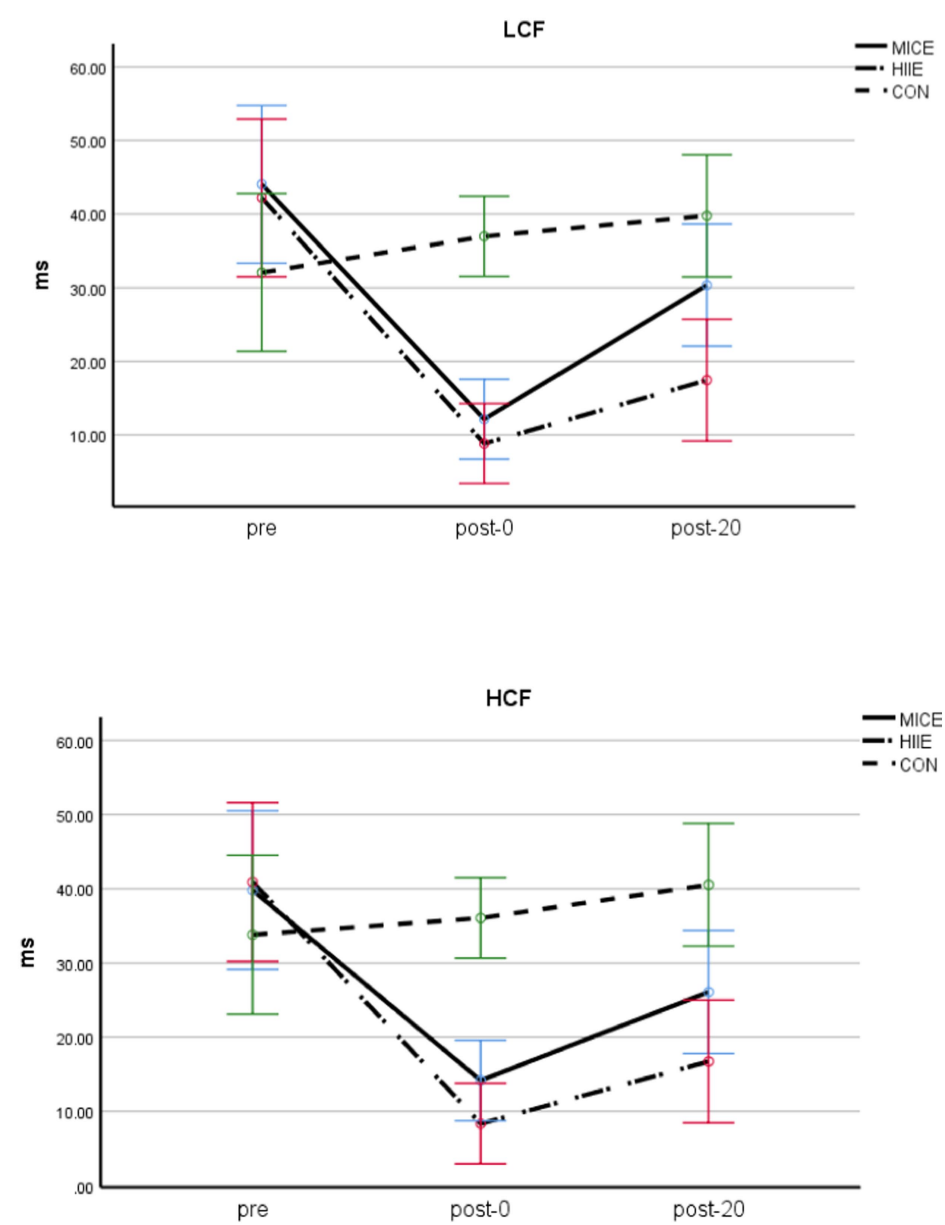
RMSSD of adolescents with obesity and different CF.

A three-factor ANOVA examining RMSSD found that the main effects of test timing [*F* (2, 83) = 84.659, *p* < 0.001, *η*_p_^2^ = 0.671] and exercise methods [*F* (2, 84) = 9.056, *p* < 0.001, *η*_p_^2^ = 0.177] were significant, and the interaction between test timing and exercise methods was also significant [*F* (4,168) = 16.732, *p* < 0.001, *η*_p_^2^ = 0.285]. Further simple effect analyses found that, for both LCF and HCF in the MICE condition, there were significant differences between pre and post-0 (*p* < 0.001), pre and post-20 (*p* < 0.01), as well as post-0 and post-20 (*p* < 0.001; post-0 > post-20 > pre). For both LCF and HCF in the HIIE condition, there were significant differences between pre, post-0, and post-20 (*p*s < 0.001; post-0 > post-20 > pre). For both LCF and HCF in the CON condition, there were no significant differences between the pre, post-0, and post-20 (*p*s > 0.05). Finally, there was no significant difference observed between HIIE and MICE before exercise (*p* > 0.05), but HIIE was significantly lower than MICE between the post-0 and post-20 (*p*s < 0.05). Other main and interaction effects were not significant (*p*s > 0.05; see Table 3 and Figure 5).

#### 3.3.2. HRV frequency domain index

**Figure 6:**
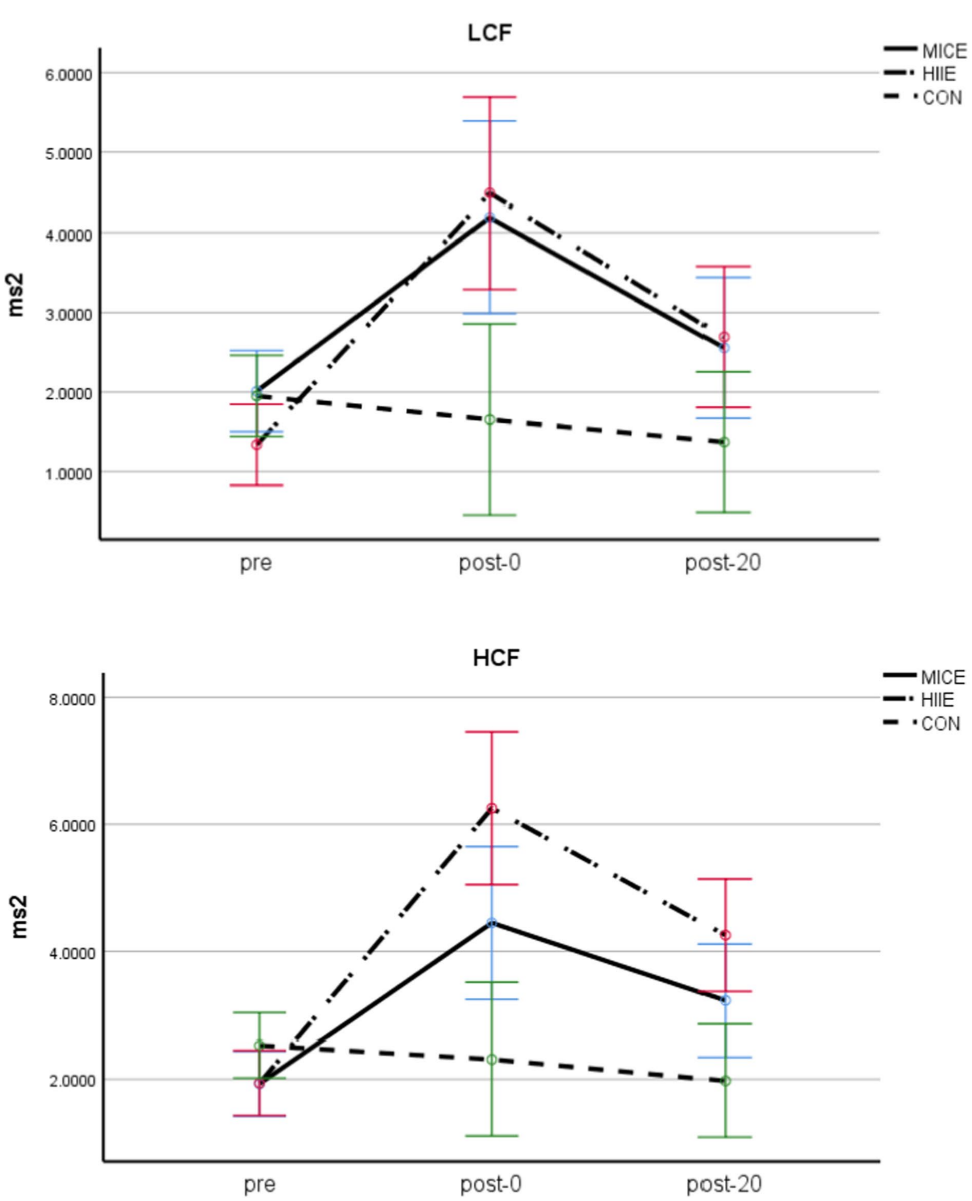
LF/HF in adolescents with obesity and different CF.

A three-way ANOVA for LF/HF found that the main effects of test timing [*F* (2, 83) = 47.221, *p* < 0.001, *η*_p_^2^ = 0.553] and exercise method (*F* (2, 84) = 9.230, *p* < 0.0001, *η*_p_^2^ = 0.180) were significant, and the interaction between test timing and exercise methods [*F* (4,166) = 11.308, *p* < 0.001, *η*_p_^2^ = 0.212] was also significant. Further simple effects analyses found that, for both LCF and HCF, in the MICE condition, there were significant differences between pre and post-0 (*p* < 0.001), pre and post-20 (*p* < 0.05), as well as post-0 and post-20 (*p* < 0.001; post-0 > post-20 > pre). For both LCF and HCF in the HIIE condition, there were significant differences between pre and post-0, pre and post-20, as well as post-0 and post-20 (*p*s < 0.001; post-0 > post-20 > pre; see Table 3). For the CON group, there were no significant differences between any two pairs of pre, post-0, or post-20 (*p*s > 0.05). The main effect of CF level was significant [*F* (1, 84) = 6.023, *p* < 0.05, *η*_p_^2^ = 0.067], with the HCF group being significantly higher than the LCF group. Other main effects and interactions were not significant (*p*s > 0.05; see Table 3 and Figure 6).

## 4. Discussion

This study primarily explored the time-course effect of acute MICE and HIIE, achieved via power cycling, on inhibitory control and HRV in adolescents with obesity and different cardiorespiratory fitness. The results showed that the cognitive effects of acute MICE and HIIE on obese adolescents after exercise were moderated by cardiorespiratory fitness. In addition, MICE and HIIE had similar effects on HRV in adolescents with obesity with different cardiorespiratory fitness and at different moments after exercise; however, the extent of the effects was different.

The first finding of this study was that the response time under the Stroop incongruent condition (i.e., inhibited response) took longer and was less accurate than those under the Stroop congruent condition (i.e., simple response), showing the typical Stroop effect (Cohen et al., 1990). In addition, the incongruent condition shows a greater decrease in response time than the congruent conditions after exercise, which indicates that the incongruent condition is more sensitive to the positive effects of acute MICE and HIIE than the congruent condition. This is consistent with the findings of Peiffer et al. (2015), who found that, after acute aerobic exercise in older women, the change in incongruent RT on the flanker task was greater than that in the congruent condition, possibly because the incongruent condition required a greater degree of executive control (Kamijo et al., 2007).

The second finding of this study is that Stroop task performance in individuals with high cardiorespiratory fitness is significantly better than in individuals with low cardiorespiratory fitness. Dupuy et al. (2015) found that individuals with higher cardiorespiratory fitness showed faster response time and greater brain oxygenation. Higher cardiorespiratory fitness is often associated with greater cerebral blood flow and cerebral perfusion (Ainslie et al., 2008) and greater utilization of relevant cognitive resources (Mekari et al., 2015), leading to better executive function performance (Huang et al., 2015).

The third finding of this study is that the time course efficiency of acute MICE and HIIE on obese adolescents is moderated by cardiorespiratory fitness. First, all participants showed significantly lower response times and higher accuracy on the Stroop task after MICE compared to before the exercise. However, it should be noted that the positive effect of MICE on individuals with high cardiorespiratory fitness lasted until 20 min after exercise, while participants with low cardiorespiratory fitness recovered Stroop performance to some extent in the delayed stage after exercise, suggesting that participants with high cardiorespiratory fitness obtained more lasting benefits after acute MICE. Similarly, Chu et al. (2015) also found that after 30 min of moderate-intensity riding, the cognition (Stroop) of high-fitness individuals was improved more than that of low-fitness individuals (elderly people). Second, cardiorespiratory fitness significantly moderated cognitive effects after HIIE. Individuals with high cardiorespiratory fitness had significantly lower response time and higher accuracy in both immediate and delayed stages after HIIE. In contrast, HIIE worsened Stroop performance (RT improved) in participants with low cardiorespiratory fitness immediately after exercise, while Stroop performance improved significantly only in inconsistent conditions at 20 min after exercise. Similarly, Cooper et al. (2018) also found that game-based high-intensity interval exercise may be particularly beneficial for adolescents with high cardiorespiratory fitness, but may be detrimental for adolescents with low cardiorespiratory fitness (Stroop and Sternberg).

The effect of exercise intensity may depend on the individual’s tolerance to the stimulation of physical activity, and cardiorespiratory fitness levels may moderate physiological demands and/or perception of exercise stimulation during exercise (American College of Sports Medicine, 2018). For a person who regularly participates in physical activity, moderate or even high-intensity exercise can be quickly adapted and completed, but for those who are inactive or sedentary, even moderate-intensity exercise may be more difficult. The current study found that HIIE worsened Stroop task performance in low cardiorespiratory fitness individuals immediately after HIIE, suggesting that acute HIIE may be too demanding for participants with low cardiorespiratory fitness. For example, Hogan et al. (2015) found that a 20 min session of moderate-intensity aerobic exercise can improve the inhibitory control (Flanker) performance of adolescents with high cardiorespiratory fitness, but reduces the inhibitory control performance of adolescents with low cardiorespiratory fitness. In contrast to individuals with low cardiorespiratory fitness, individuals with high cardiorespiratory fitness have more lasting benefits after acute MICE and HIIE, possibly because individuals with high cardiorespiratory fitness were accustomed to greater metabolic load and thus had fewer exercise task constraints. Thus, more resources are allocated to cognitive tasks (Brisswalter et al., 2002). Meanwhile, the enhancement of brain lactic acid metabolism caused by high-intensity exercise may also be another possible reason for the improvement of HIIE’s extended executive function (Tsukamoto et al., 2016).

It is also important to note that Gejl et al. (2018) argued that high cardiorespiratory fitness was not a prerequisite for temporary improvements in executive function, as participants with low and high cardiorespiratory fitness seemed to benefit to the same extent (Ludyga et al., 2016). Drollette et al. (2014) also believe that individuals with high cardiorespiratory fitness are more likely to have a ceiling effect in cognitive assessment, while individuals with low cardiorespiratory fitness may have more room for improvement after a single exercise. As summarized in an analysis by Brisswalter et al. (2002), there are conflicting results regarding the effect of cardiorespiratory fitness on cognitive performance during or after acute exercise; thus, further research will contribute to a better understanding of the role of cardiorespiratory fitness in cognitive improvement after acute exercise.

The fourth finding of this study was that MICE and HIIE produced similar but different time course efficiency on HRV in obese adolescents with different cardiopulmonary fitness. Compared with the control group, RMSSD was significantly reduced and LF/HF were significantly increased in all participants in the immediate and delayed stages after MICE and HIIE, but the degree of influence of HIIE on HRV-related indicators was greater than that of MICE, indicating that HIIE induced lower HRV and higher arousal level after exercise compared with MICE. During exercise, sympathetic nerve activity increases and parasympathetic nerve activity decreases. This autonomic regulation of cardiac activity is modulated by exercise intensity; the greater the intensity of exercise, the more sympathetic and less parasympathetic nervous system activation (Kaikkonen et al., 2010), the lower the HRV after exercise (Kaikkonen et al., 2007). The shift from balanced HRV to sympathetic dominance reflects increased arousal levels that contribute to executive function (Kashihara et al., 2009).

In addition, changes in RMSSD and LF/HF in high cardiorespiratory fitness individuals were greater than those in low cardiorespiratory fitness individuals in the immediate and delayed stages after MICE and HIIE, indicating that exercise-induced arousal levels in high cardiorespiratory fitness individuals were higher than those in low cardiorespiratory fitness individuals. However, it is important to note that at the delayed phase after exercise, arousal levels of the low cardiorespiratory fitness individuals recovered somewhat, but arousal levels of the high cardiorespiratory fitness individuals remained at higher levels, which may be one reason why the individuals with high cardiorespiratory fitness experienced more lasting benefits after acute MICE and HIIE. The study also found that Stroop task performance in individuals with low cardiorespiratory fitness was improved (decreased RT) immediately after MICE, but worsened (increased RT) immediately after HIIE. According to the inverted U hypothesis (Yerkes and Dodson, 1908), if MICE induces the appropriate arousal level for cognitive performance in the low cardiorespiratory fitness group after exercise, However, the arousal induced immediately after HIIE exercise may exceed the appropriate arousal level to induce positive cognitive effects, resulting in decreased behavioral performance.

The current study has some limitations. First of all, due to the particularity of the subject group, difficulties in the selection of subjects such as the opposition from parents, and the loss of subjects in the exercise intervention, the number of subjects in this study is finally small. In addition, the selection criteria for obese adolescents are lower than international standards, which may affect the interpretation of the more real results of the study. Second, based on the safety of the exercise intervention of the subjects, the submaximal intensity YMCA cycle ergometer test program was selected to complete the cardiorespiratory fitness test, instead of using the more standard gas analysis test method, which may have a certain impact on the division of cardiorespiratory fitness groups. Third, due to the short-term HRV test of 5 min, the Stroop task test immediately after exercise was delayed by 5 min, which may affect the judgment of the subjects’ cognitive effects immediately after exercise. Fourth, this study only investigated the temporal effects immediately and at 20 min after acute MICE and HIIE, not the longer duration, the duration of cognitive effects after acute MICE and HIIE remains unknown. Therefore, based on the above limitations, further research is needed on the acute exercise-cognition relationship.

## 5. Conclusion

The current study found that the acute effects of MICE and HIIE on inhibitory control in obese adolescents were influenced by the interaction of cognitive test timing and cardiorespiratory fitness. Individuals with high cardiorespiratory fitness performed better on the Stroop task than individuals with low cardiorespiratory fitness. The inhibitory control of HIIE in high-cardiorespiratory obese adolescents produced positive effects similar to those in MICE but more lasting, suggesting that HIIE is more beneficial for high-cardiorespiratory obese adolescents. MICE promoted inhibitory control in obese adolescents with low cardiorespiratory fitness, but HIIE impaired inhibitory control in obese adolescents with low cardiorespiratory fitness immediately after exercise, suggesting that low cardiopulmonary fitness obese adolescents may be suitable for MICE rather than HIIE exercise intervention. Therefore, it is recommended that obese adolescents consider their cardiorespiratory fitness level when choosing the type of exercise that is the most time efficient and maximizes cognitive ability. In addition, the study found that the shift from balanced HRV to sympathetic dominance after acute exercise reflects increased arousal levels and may be one of the underlying mechanisms by which acute exercise brings benefits to executive function.

## Data availability statement

The raw data supporting the conclusions of this article will be made available by the authors, without undue reservation.

## Ethics statement

The studies involving humans were approved by the Ethics Committee of Beijing Sport University. The studies were conducted in accordance with the local legislation and institutional requirements. Written informed consent for participation in this study was provided by the participants’ legal guardians/next of kin.

## Author contributions

Z-TF: research design, data analysis, and writing. Z-XM: writing—review and editing. F-BL: designing computer programs and data curation. X-WO: experiment implementation and data collection. All authors contributed to the article and approved the submitted version.

## Conflict of interest

The authors declare that the research was conducted in the absence of any commercial or financial relationships that could be construed as a potential conflict of interest.

## Publisher’s note

All claims expressed in this article are solely those of the authors and do not necessarily represent those of their affiliated organizations, or those of the publisher, the editors and the reviewers. Any product that may be evaluated in this article, or claim that may be made by its manufacturer, is not guaranteed or endorsed by the publisher.
